# Home-based end-of-life care for people with dementia: A systematic review of quantitative and qualitative evidence

**DOI:** 10.1177/14713012241308625

**Published:** 2024-12-15

**Authors:** Guo Yin, Leah Macaden, Divya Sivaramakrishnan, Yajing Wang, Lian Zhu, Huimin Chong

**Affiliations:** Nursing Studies, School of Health in Social Science, 3124 University of Edinburgh, Edinburgh, UK

**Keywords:** home, end-of-life care, hospice care, palliative care, dementia, review

## Abstract

**Background:** Integrating home-based end-of-life care for people with dementia will become increasingly important as the population ages. Therefore, it is timely and necessary to evaluate the evidence of home-based end-of-life care for people living with dementia. **Aim: **This review aims to identify the characteristics of home-based end-of-life care interventions for people living with dementia and review the existing evidence on implementation outcomes. **Design:** Systematic Review and Narrative Synthesis. The Mixed Methods Appraisal Tool was used to assess study quality. **Data sources:** A comprehensive search was conducted across five databases (PubMed, Web of Science, MEDLINE CINAHL and Scopus) from June to August 2023, and the citations to the included studies were tracked through citation tracking in Google Scholar to identify potentially relevant studies. **Results:** Of the 2022 articles retrieved, 12 were included in this review. The included studies were geographically diverse, with four from the United States, three from Singapore, two from the United Kingdom, and one each from the Netherlands, Belgium, and Israel. Additionally, due to the difference of focus and nature of the studies, only seven of these studies provided information on home-based end-of-life care interventions for people living with dementia. The interventions identified in this review align closely with the essential components of optimal palliative care for dementia outlined in the European Association for Palliative Care white paper. However, the evidence supporting these home-based end-of-life care interventions for people living with dementia is constrained by the number of studies and methodological limitations. Nevertheless, this systematic review still identifies some evidence supporting home-based end-of-life care for people living with dementia, including reduced healthcare utilization and costs, as well as help people living with dementia realize their wish to die at home. **Conclusions:** Whilst current evidence highlights benefits of home-based end-of-life care for people living with dementia, the relatively limited number, methodology of studies, the heterogeneity of study focus and outcome measures hinder the formation of definitive conclusions. Therefore, further research is needed to develop and evaluate home end-of-life care services for people living with dementia.

## Introduction

Dementia is defined as a cognitive impairment that severely affects activities of daily living and leads to loss of independence (Pasquini et al., 2018; van der Steen, 2010). Moreover, the development of dementia is usually progressive and irreversible, getting worse over time and eventually leading to severe disability in older adults (Sousa et al., 2009, 2010). Therefore, dementia is considered one of the most burdensome diseases (Europe, 2019). Dementia is also emerging as a serious health challenge globally, with the number of people living with dementia estimated to be over 55 million worldwide (Organization, 2022) with projections that the number of people living with dementia will reach approximately around 152.8 million globally by 2050 (Nichols et al., 2022).

Dementia is associated with shorter life expectancy, making it the fifth leading cause of death globally, responsible for a staggering number of deaths each year (Scheltens et al., 2021). People with dementia are often accompanied by other comorbidities that increase their risk of death, such as impaired consciousness, pain, aspiration pneumonia, and eating disorders (Davies et al., 2021; Hum et al., 2018; Sampson et al., 2018). This also exacerbates patients’ suffering, which makes them more vulnerable in the final stages of life. Considering this predicament, end-of-life care has become a pressing issue.

People with dementia may benefit from palliative and end-of-life care, even though there is no reversible therapy for dementia illness (Piovezan et al., 2020). Palliative and end-of-life care is comfort-oriented, preventing and alleviating patients’ suffering through early treatment of pain, addressing other physical, mental and spiritual issues, and maintaining the highest quality of life until death, rather than merely prolonging life (Organization, 2018; Radbruch et al., 2020; Van der Steen et al., 2014). There are several venues where end-of-life care can be provided, including homes, nursing homes, and hospitals (Wang & Wang, 2020). However, most people express a preference for receiving end-of-life care at home (S. Shepperd et al., 2021). This is because one’s own home provides familiarity, comfort, and a more intimate atmosphere than the hospital environment, which helps patients to feel accompanied and cared for by their families in the last days of their lives (Wahid et al., 2018). This comfortable environment not only reduces patients’ anxiety and distress but also contributes to improving their quality of life (Gozalo et al., 2011). Consequently, the home is becoming the preferred place for an increasing number of terminally ill patients as it helps them to live the last stages of their lives with dignity and meaning (Higginson & Sen-Gupta, 2000).

The integration of home end-of-life care program into dementia care is a first step towards alleviating end-of-life suffering and improving the quality of death and is a key factor in helping people with dementia to achieve a ‘good death' (Hoare et al., 2015). However, despite the many advantages of home end-of-life care, which have been favorably viewed in several studies (Brumley et al., 2007; Gomes et al., 2016), current evidence shows mixed findings on the ultimate practical outcomes of end-of-life care at home, particularly as some studies have found that home end-of-life care is associated with long hospice stays (>180 days) and live discharges from hospice (Luth et al., 2020, 2021). This issue is highlighted in people living with dementia. As dementia progresses, the symptom burden and physical dependency of people with dementia increases, requiring high levels of care that are often beyond the capacity of their family caregivers (Peacock, 2013). This can lead to hospitalization, most commonly in a nursing home. It also results in people living with dementia rarely dying at home (Sleeman et al., 2014). They are more likely to die in a hospital or long-term care facility (Reyniers et al., 2015). Therefore, although one’s own home may be a satisfactory end-of-life care setting for people living with dementia, unfortunately, the lack of evidence on home-based end-of-life care for this population leaves it unclear whether home can ultimately serve as their final place of care (Miranda et al., 2019).

### Aim

This review aimed to assess and identify current evidence on home-based end-of-life care for people living with dementia by synthesizing findings from quantitative and qualitative studies, as well as to identify key gaps in the existing evidence that still need to be addressed more comprehensively.

### Objectives

Specifically, the purpose of this review was to identify existing evidence on (i) interventions, outcomes, and effectiveness, including cost-effectiveness, of home-based end-of-life care for people living with dementia, and (ii) the experiences of people living with dementia and their family caregivers who utilize home-based end-of-life care services, as well as the perspectives of those who provide such care services.

## Method

This review used a combination of quantitative and qualitative data (Thomas et al., 2004), and was conducted in strict adherence to the Preferred Reporting Items for Systematic Reviews and Meta-Analyses (PRISMA) statement (Moher et al., 2009).

### Eligibility criteria

Inclusion and exclusion criteria were determined using participant, intervention, outcome, and study design methods. Only studies that met the following criteria were eligible for inclusion.

#### Participants

Participants with dementia, including all types of dementia or family members or informal caregivers, or healthcare professionals involved in providing end-of-life care for people living with dementia.

#### Interventions

Studies evaluating end-of-life care services provided in the home setting were included in this review. For the purposes of the review of evidence on home-based end-of-life care, interventions were defined as any type of data about end-of-life care services or interventions at home for people with dementia. End-of-life care is part of palliative care and includes managing symptoms as well as providing psychological, social and spiritual support and ensuring that the wishes of the patient and family are followed (Alshammari et al., 2022). Therefore, studies assessing home-based end-of-life care, home hospice or home palliative care were included in the review. Studies of palliative or end-of-life care in nursing home settings or in the community, or in hospitals were excluded.

#### Outcomes

Studies encompassing one or a broad range of outcomes related to home-based end-of-life care associated with people living with dementia, family caregivers, healthcare professionals, including, but not limited to, quality of life, quality of death, dementia symptoms, comfort, place of death, satisfaction with care, health care utilization, cost of care, and survival, experience and satisfaction of care were included. Studies were excluded if the outcomes related to the needs of home-based end-of-life care for people living with dementia.

#### Study design

Randomized and non-randomized controlled trials, non-comparative trials, and qualitative or mixed-methods studies were included in the study. Case reports, conference proceedings and books, literature reviews and unpublished and grey literature were excluded.

### Search strategy

Recognizing that relatively few studies were likely to be found, and to reduce the risk of missing studies, the search terms covered only the following three domains: (i) end-of-life care, (ii) dementia, and (iii) home. No specific terms were used in the search to describe study designs, interventions, comparison groups, or outcomes. The search was conducted from June to August 2023, and 5 electronic databases (PubMed, Web of Science, MEDLINE, CINAHL and Scopus) were searched. The search strategy was adapted to the different databases. The researcher (GY) also searched the reference lists of the included studies to identify other relevant articles. Additionally, citations to the included studies were tracked through citation tracking in Google Scholar. The full text of all identified potentially relevant citations was manually reviewed to verify that they met the inclusion criteria. Additional language restrictions were used to include only English-language publications, but no geographic or year restrictions were set.

Review articles were uploaded to Endnote reference management software (Gotschall, 2021) and duplicate studies were removed. One researcher (GY) initially screened titles and abstracts and excluded irrelevant studies. Next, two reviewers (GY & YW) independently screened the full texts of relevant studies. Studies that did not meet the inclusion criteria were deleted, and the reasons for exclusion were recorded. Discrepancies between reviewers regarding inclusion were resolved through discussion.

### Data extraction

Data extraction forms were used to extract data for evidence synthesis and to assess study quality. Data extraction and analysis were carried out in stages. Data were first extracted independently by one researcher (GY) according to a pre-designed standardized data extraction form. The data extraction tool was based on the Cochrane checklist (Higgins & Green, 2008). The second researcher (HC) then verified and checked the data in the table and resolved any differences through discussion.

### Data synthesis, and analysis

Due to the high degree of heterogeneity in the studies included relating to outcomes, study design and participants, neither meta-synthesis of qualitative studies nor meta-analysis of quantitative studies was applicable. Due to this situation, a narrative synthesis analysis approach was adopted, which offers flexibility in combining quantitative and qualitative data (Lipsey & Wilson, 2001).

Using the EAPC White Paper’s guidelines for optimal palliative care for people with dementia as a framework (Van der Steen et al., 2014), this review synthesized the key points of the included interventions to determine whether existing home-based end-of-life care practices meet the latest standards of best practice in palliative and hospice care for people with dementia.

### Quality assessment

In this systematic review, the Mixed Methods Appraisal Tool (MMAT) (version 2018), which is a validated tool and allows for the assessment of studies of a wide range of designs, was used due to the combined review of quantitative and qualitative methodological studies (Hong et al., 2018). Quality assessment was conducted independently by two reviewers GY & LZ. Disagreements between the two reviewers were settled by discussion until agreement was reached. It is important to emphasize that no study was excluded from this systematic review based on quality assessment.

## Results

### Study selection

**Figure 1.**
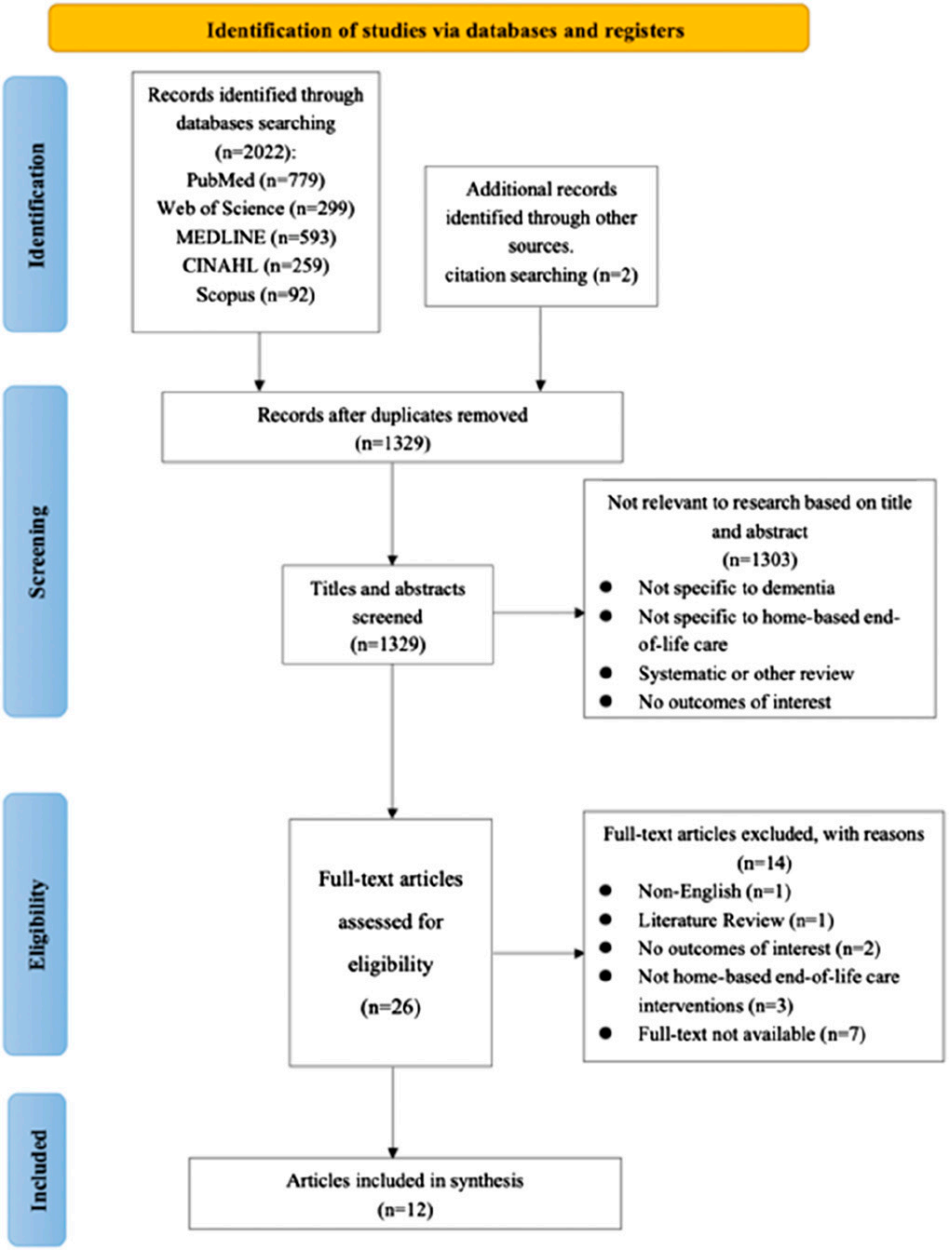
PRISMA flow chart of literature search (Moher et al., 2009

A total of 2022 studies were identified from the electronic database search. After removing 693 duplicates, a total of 24 studies were considered relevant after title and abstract screening. Fourteen studies were excluded after full-text screening. Reasons for exclusion included non-relevance to the interventions and outcomes on home-based end-of-life care for people living with dementia, non-full text or non-English and review articles. Finally, two relevant studies were identified in a citation search of 10 studies that met the inclusion criteria. The final 12 studies were included in this systematic review (Bolt et al., 2019; Brian Cassel et al., 2016; Holley et al., 2009; Hum et al., 2020; Luth et al., 2021; Miranda et al., 2021; Mitchell et al., 2004; Mogan et al., 2022; Pereira et al., 2023; Sternberg et al., 2019; Tay et al., 2020; Treloar et al., 2009). Detailed study screening, inclusion and exclusion processes are shown in Figure 1.

### Study characteristics and quality

All 12 studies were published between 2004 and 2023. Eight studies were quantitative studies, of which two were prospective cohort (Hum et al., 2020; Tay et al., 2020) and five were retrospective cohort studies (Brian Cassel et al., 2016; Luth et al., 2021; Miranda et al., 2021; Mitchell et al., 2004; Pereira et al., 2023); with one pilot study with a pre-post design (Sternberg et al., 2019). In addition, three studies were qualitative studies (Bolt et al., 2019; Mogan et al., 2022; Treloar et al., 2009) and one study was a mixed method study (Holley et al., 2009).

The included studies have a diverse international distribution, with four studies conducted in the United States (Brian Cassel et al., 2016; Holley et al., 2009; Luth et al., 2021; Mitchell et al., 2004), and three studies from Singapore (Hum et al., 2020; Pereira et al., 2023; Tay et al., 2020), two from the United Kingdom (Mogan et al., 2022; Treloar et al., 2009), one each from the Netherlands, Belgium, and Israel (Bolt et al., 2019; Miranda et al., 2021; Sternberg et al., 2019).

Regarding the participants, seven studies mainly focused on people living with dementia (Hum et al., 2020; Luth et al., 2021; Miranda et al., 2021; Mitchell et al., 2004; Pereira et al., 2023; Sternberg et al., 2019; Tay et al., 2020), and two studies included people with terminal illness (Brian Cassel et al., 2016; Holley et al., 2009), of whom living with dementia accounted for 64% and 25.5% respectively; Three studies included family caregivers as study participants (Bolt et al., 2019; Mogan et al., 2022; Treloar et al., 2009). In addition, two studies focused on comparing the experience of end-of-life care for people with dementia in nursing homes and home care facilities (Bolt et al., 2019; Mitchell et al., 2004). Characteristics of the 12 studies are detailed in Table 1. The quality of included studies was variable, but overall the studies included are of good methodological quality. The specific quality appraisal results are shown in Table 2.

**Table 1.** Details of included studies.

Authors, year, country	Study design	Sample demographics	Aims	Intervention	Outcome measures	Key findings/ Results	Main conclusion
Bolt et al. (2019) The Netherlands	Qualitative study	Relatives of people with dementia who had experienced end-of-life care in nursing home or home care (*n* = 32), people with advanced dementia receiving home care (*n* = 8)	To investigate relatives’ experiences with end-of-life care for people with dementia, comparing the nursing home and home setting	The desired dementia care towards end-of-life project carried out in the living lab in ageing and long-term care	Relatives’ experiences with end-of-life care for people with dementia	The relatives of individuals who were receiving home care mostly spoke of satisfying collaboration with nursing staff and mentioned that the nursing staff were focused on individualized care; Relatives being emotionally supported and being involved, informed, and heard	Person-centered care was not as apparent in the nursing home setting, as it was to the relatives of someone being cared for at home. Relatives appreciated their caregiving role more compared to relatives of someone who was being cared for at a nursing home
Mogan et al. (2022) The United Kingdom	Qualitative study	Informal caregivers who had looked after a person with dementia at home (*n* = 29)	To explore informal caregivers’ views and experiences of health and social care services when looking after a person with dementia at home at the end-of-life	Non-specialist home-based end-of-life care	Informal caregivers’ experiences	Poor continuity of care; lack of expertise; limited advance care planning; and loss of autonomy	While more people are dying at home with dementia, this increase has not been matched by an increase in resource, infrastructure, staff, and capacity. Specialist palliative care for people with dementia is rare and mostly managed by GPs and domiciliary care workers. Additionally, domiciliary care services are failing to deliver end-of-life care that is personalized
Treloar et al. (2009) The United Kingdom	Qualitative study	Caregivers of people with dementia (*n* = 14)	To undertake detailed interviews of key caregivers who had supported people with dementia at home, with the aim of identifying the major factors which make home-based and palliative care feasible	Palliative and end of life care of dementia at home	Family members’ experiences	***Funding:*** Home-based end of life care saved statutory services large amounts of money. The overall cost of care was lower than dementia nursing care ***Grief:*** The extra work and involvement of hands-on care for someone with dementia may alleviate rather than simply delay that grief	Managing advanced dementia through till death at home is feasible and good, home-based palliative care of dementia can be achieved with very positive outcomes
Brain Cassel et al. (2016) The U.S.	Observational, retrospective study	Individuals who received the home-based palliative care (*n* = 368), people with dementia (*n* = 92) Comparison: No received the home-based palliative care (*n* = 1075), people with dementia (*n* = 276)	To evaluate the nonclinical outcomes of a proactive palliative care program funded and operated by a health system for medicare advantage plan beneficiaries	Home- and clinic-based palliative care services (transitions program)	Healthcare use; costs	Intervention participants in all four disease groups had less hospital use and lower hospital costs nonintervention participants, which drove lower overall healthcare costs. In the final 6 months of life, healthcare costs for the intervention groups stayed largely the same from month to month, whereas costs for comparison participants increased dramatically	In the context of an alternative payment model in which the provider was “at risk” of bearing the costs of care, a proactive palliative care program helped to avoid the escalation in hospital use and costs commonly seen in the final months of life
Hum et al. (2020) Singapore	A prospective cohort study	People with dementia (*n* = 254)	This study explores patients’ symptoms and quality-of-life and their association with enteral feeding, evaluates the impact of an integrated palliative homecare program for advanced dementia on these parameters and examines familial caregiver burden	An integrated homecare program	Patients’ symptoms and quality-of-life; caregiver burden	***Patients’ symptoms and quality-of-life***: Median NPI-Q severity scores reduced from 3 (1-9) to 1 (0-3), *p* = .001; median QUALID scores reduced significantly from 23 (IQR 19-30) to 19 (IQP 15-24), *p *= .001 ***Caregiver burden***: Median ZBI score for caregivers was 26 (IQR 15–36). Having stay-in help reduced it from 39.5 (IQR 25–49) to 25 (IQR 15–35), *p* = .001	An integrated multidisciplinary palliative homecare team with geriatric training that is accessible all-hours addressed the needs of home-dwelling people with advanced dementia, improved their quality-of-life and supported families to care for them at home
Luth et al. (2021) The U.S.	Retrospective cohort study	People with dementia who received hospice services (*n* = 3837); 80% received home hospice (*n* = 3085)	Examine how home hospice and nurse visit frequency relate to dying in hospice within the medicare-intended 6-month period	Patients who received hospice care from a large not-for-profit hospice agency	Death within 6 months; live discharge or long length of stays	Home hospice patients were more likely to experience live discharge or long length of stays (HR for death: 0.77, 95%CI: 0.69–0.86, *p* < .001); Patients who received home hospice had a 23% lower hazard of expected death (adjusted hazard ratio (AHR): 0.77; 95%CI: 0.69, 0.86; *p* < .001), compared to patients who received hospice in nursing homes; Predicted probability of survival is greater for home hospice patients than for nursing home patients. At 30 days of hospice, home hospice patients have a 0.83 predicted probability of survival, compared to 0.81 for nursing home patients	People with dementia who receive home hospice care, compared to in nursing homes, were less likely to experience an expected death, increasing their risk for live discharge, and potentially resulting in uncertainty and distress when hospice services are withdrawn Nurse visits reduce risk of these outcomes
Miranda et al. (2021) Belgium	Propensity-matched decedent cohort study	Home-dwelling older people who died with dementia (*n* = 23,670)	To evaluate the effects of palliative home care on quality and costs of end-of-life care for older people with dementia	Palliative home care measures include home visits by a multidisciplinary palliative home care team	Costs; Place of death; health services utilization	Those using palliative home care support, in the last 14 days of life, had lower risk of hospital admission (17.5% vs. 50.5%; relative risk (RR) = 0.21), undergoing diagnostic testing (17.0% vs. 53.6%; RR = 0.20) and receiving inappropriate medications, but were more likely to die at home (75.7% vs. 32.6%; RR = 6.45) and to have primary care professional contacts (mean 11.7 vs. mean 5.2), compared with those who did not. Further, they had lower mean total costs of care in the last 30 days of life	Palliative home care use by home-dwelling older people with dementia is associated with improved quality and reduced costs of end-of-life care
Mitchell et al. (2004) The U.S.	Retrospective cohort study	People with advanced dementia in nursing home (*n* = 2730) People with advanced dementia receiving home care services (*n* = 290)	To describe and compare the end-of-life experience of persons dying with advanced dementia in the nursing home and home care settings	Patients receive terminal care in nursing homes, others remain in the community with home care services	Health services utilization; nonpalliative interventions; signs and symptoms; treatments	***Health services utilization:*** There was no difference in hospitalization rates between nursing homes and home care clients, but hospice referral was significantly lower among nursing home residents compared to home care clients ***Nonpalliative interventions:*** Patients living in nursing homes were more likely to receive non-palliative care than those with dementia who received home care. For example, among nursing home residents with advanced dementia, 27.2% died with a feeding tube, compared to 11.9% of subjects in the home care program (*p* < .001) ***Signs and symptoms***: Compared to home care clients, subjects in the nursing home were reported to have less pain (37.1 % vs. 53.4%, *p* < .001) and shortness of breath (12.7% vs. 29.7%, *p* < .001). Pneumonia was also significantly more likely among the nursing home residents. The likelihood of depression did not differ between the two groups. Other treatments ***Treatments:*** Compared to home care clients, subjects were more likely to receive oxygen therapy if they resided in the nursing home (24.4% vs. 12.5%, *p* < .001)	Compared to home care, several palliative care outcomes in advanced dementia may be more favorable in the nursing home. Reported pain and shortness of breath were less common in the nursing home subjects compared to home care cohort, whereas advance directives and the use of oxygen therapy were more common in the nursing home setting. However, palliative care was not optimal in either setting
Pereira et al. (2023) Singapore	Retrospective cohort study	Home-dwelling patients with advanced dementia (program Dignity = 184, controls = 139)	To determine the economic benefit of an integrated home-based palliative care program for advanced dementia (program dignity), evaluation is required	An integrated home-based palliative care program for advanced dementia (program dignity)	Healthcare utilization; costs	The average monthly program cost was SGD$1311 (SGD-pounds exchange rate: 0.481) per patient. Fully enrolled program patients were less likely to visit the emergency department (incidence rate ratios (IRRs): 1 month = 0.56; 3 months = 0.19; 6 months = 0.10; all *p* < .001), be admitted to hospital (IRRs: 1 month = 0.60; 3 months = 0.19; 6 months = 0.15; all *p* < .001), had a lower cumulative length of stay (IRRs: 1 month = 0.78; 3 months = 0.49; 6 months = 0.24; all *p* < .001) and incurred lesser healthcare utilization costs (β-coefficients: 1 month = 0.70; 3 months = 0.40; 6 months = 0.43; all *p* < .01) at all time-points examined	Geriatric palliative homecare model for people with advanced dementia enables them to be cared for at home whilst reducing healthcare utilization and costs
Sternberg et al. (2019) Israel	Quasi-experimental study, prospective pilot pre-post, single group design	Older with advanced dementia receiving home care services (*n* = 20)	To examine clinical and health services outcomes of a quality improvement pilot project to provide home hospice care for older people with advanced dementia	Maccabi healthcare services homecare program	Symptom management; satisfaction with care; caregiver burden	***Symptom management:*** Score of 33.8 on admission on the volicer symptom management scale increased to 38.3 on discharge ***Satisfaction with care****:* scores increased from 27.5 to 35.3 ***Caregiver burden:*** scores dropped from 12.1 to 1.4 on the zarit burden index	The current quality improvement pilot project of adding hospice care to usual homecare showed improved symptom management and increased satisfaction of family caregivers with care, reduction in caregiver burden and in hospitalizations, and discontinuation of unnecessary medications
Tay et al. (2020) Singapore	A prospective cohort study	Patients from the palliative homecare program (*n* = 297)	To identify modifiable factors associated with the comfort of people with dementia dying at home and families’ satisfaction with care	Palliative homecare program	Comfort at the end of life; satisfaction with care	***Comfort at the end of life***: Median CAD-EOLD scores increased from 37 (34–40) to 38 (36–40) and the mean increased from 36.5 (4.2) to 37.8 (3.2), *p* = .001 from baseline to death. Specifically, the mean scores on the emotional distress and well-being subscales increased from 11.4 (1.1) to 11.8 (0.6), *p* < .0001 and from 7.5 (1.8) to 8.0 (1.6), *p* = .007, respectively. There was no significant change on the physical distress and dying symptoms subscales ***Satisfaction with care***: About 85% of family caregivers were interviewed with SWC-EOLD two months after bereavement. Median SWC-EOLD score was 38 (34–40) and mean was 36.5 (3.8)	Achieving comfort and satisfaction with care for people with dementia dying at home involves an interplay of modifiable factors. Honoring medical intervention preferences, such as those with palliative intent associated with patients’ comfort, determined families’ satisfaction with care
Holley et al., (2009) The U.S.	Mixed-methods study (chart review, telephone interviews, and face-to-face interviews)	Chart review: Patients enrolled in PATCH (*n* = 74), 64% people with dementia Face–face interview: Caregivers (*n* = 13) Telephone interviews: Caregivers (*n* = 22)	The objective was to better understand how caregivers of patients who died defined a successful home palliative care experience and to use these definitions to improve care and help avoid terminal hospitalization in cases in which home death was preferred, the predominant preference of most patients	Palliative access through care at home program	**Quantitative:** Place of death; satisfaction **Qualitative:** Location of care; access and expertise; transitions of care	**Quantitative results:** ***Place of death***: More than two-thirds of PATCH patients died at home or in an inpatient hospice setting ***Satisfaction***: Amount of care required and satisfaction with PATCH were generally high **Qualitative results**: PATCH appeared to help some patients stay at home and avoid institutionalization; ease of access to a geriatrics and palliative care expert; help caregivers with care transitions	PATCH helped to ease the transition between acute, hospital-based care and home-based end-of-life care

Abbreviations*:* NPI-Q: the Neuropsychiatric Inventory Questionnaire; QUALID: the Quality of Life in late-stage Dementia tool; ZBI: the Zarit Burden interview; CAD-EOLD: the Comfort Assessment in Dying with Dementia; SWC-EOLD: the Satisfaction with Care at the End-of-Life in Dementia; PATCH: Palliative Access Through Care at Home.

**Table 2.** MMAT quality appraisal results.

Studies	Screening questions		1.Qualitative					Score
	S1. Are there clear research questions?	S2. Do the collected data allow to address the research questions?	1.1. Is the qualitative approach appropriate to answer the research question	1.2. Are the qualitative data collection methods adequate to address the research question?	1.3. Are the findings adequately derived from the data?	1.4. Is the interpretation of results sufficiently substantiated by data?	1.5. Is there coherence between qualitative data sources, collection, analysis and interpretation?	
Bolt et al. (2019) The Netherlands	Y	Y	Y	Y	Y	Y	Y	100%
Mogan et al. (2022) The UK	Y	Y	Y	Y	Y	Y	Y	100%
Treloar et al. (2009) The UK	Y	Y	Y	Y	C	C	Y	60%

Studies	Screening questions		3. Quantitative non-randomized studies					Score
	S1. Are there clear research questions?	S2. Do the collected data allow to address the research questions?	3.1. Are the participants representative of the target population?	3.2. Are measurements appropriate regarding both the outcome and intervention (or exposure)?	3.3. Are there complete outcome data?	3.4. Are the confounders accounted for in the design and analysis?	3.5 during the study period, is the intervention administered (or exposure occurred) as intended?	
Brain Cassel et al. (2016) The U.S.	Y	Y	Y	Y	Y	Y	Y	100%
Hum et al. (2020) Singapore	Y	Y	Y	Y	Y	C	Y	80%
Luth et al. (2021) The U.S.	Y	Y	Y	Y	Y	Y	Y	100%
Miranda et al. (2021) Belgium	Y	Y	Y	Y	Y	Y	Y	100%
Mitchell et al. (2004) The U.S.	Y	Y	Y	Y	Y	Y	Y	100%
Pereira et al. (2023) Singapore	Y	Y	Y	Y	Y	Y	Y	100%
Sternberg et al. (2019) Israel	Y	Y	Y	Y	Y	C	Y	80%
Tay et al. (2020) Singapore	Y	Y	Y	Y	Y	Y	Y	100%

Studies	Screening questions		5. Mixed methods					Score
	S1. Are there clear research questions?	S2. Do the collected data allow to address the research questions?	5.1. Is there an adequate rationale for using a mixed methods design to address the research question?	5.2. Are the different components of the study effectively integrated to answer the research question?	5.3. Are the outputs of the integration of qualitative and quantitative components adequately interpreted?	5.4. Are divergences and inconsistencies between quantitative and qualitative results adequately addressed?	5.5. Do the different components of the study adhere to the quality criteria of each tradition of the methods involved?	
Holley et al. (2009) The U.S.	Y	Y	Y	Y	Y	Y	Y	100%

*Note.* *Y = YES, *N* = NO, C = Cannot; MMAT: Mixed Methods Appraisal Tool (version 2018). 1. Qualitative; 2. Quantitative randomized controlled trials; 3. Quantitative nonrandomized; 4. Quantitative descriptive; 5. Mixed methods.

### Description of home-based end-of-life care interventions

Home-based end-of-life care services and interventions for people living with dementia in certain studies lacked clear definitions, especially in qualitative and retrospective studies. This can be attributed to differences in the nature and focus of studies. Qualitative studies prioritize subjective experiences and perspectives (Bolt et al., 2019; Mogan et al., 2022; Treloar et al., 2009), Retrospective studies use pre-existing data from patient electronic medical records and databases, which may have obscured intervention specifics (Luth et al., 2021; Mitchell et al., 2004). Consequently, while these studies all examined home-based end-of-life care for people living with dementia, some emphasized detailed intervention descriptions, while others explored various outcomes and effects.

As a result, only seven studies reported dementia-related home-based end-of-life care interventions (Brian Cassel et al., 2016; Holley et al., 2009; Hum et al., 2020; Miranda et al., 2021; Pereira et al., 2023; Sternberg et al., 2019; Tay et al., 2020) (see Table 3). The care services and interventions of these studies presented some common features. First, these studies centered on multidisciplinary teams, which are trained professional hospice care teams composed of doctors, nurses, psychiatric care providers and social workers, aiming to provide comprehensive end-of-life care support for people living with dementia. These home-based end-of-life care interventions were usually based on home visits, supplemented by telephone contact, and the number of home visits were based on the needs of the patient. These studies also showed consistency in the areas of person-centered care, communication, and shared decision making, and family care and participation according to the EAPC white papers optimal palliative care practice domain for people living with dementia. However, due to the diversity of studies in their choice of intervention strategies, not all domains of optimal palliative care for people with dementia were being consistently met (see Table 4).

**Table 3.** Key components of home-based end-of-life care services (*n* = 7).

Author	Care models	Referral pathway	Care service activities and procedures	Providers	Funding
Brain Cassel et al. (2016)	Transitions program	Medical records data and billing and claims data	**Intervention focus**	A trained specialty palliative care team comprising doctors, nurses, spiritual care providers, and social workers	The program focuses on medicare advantage beneficiaries seen in medical groups affiliated with sharp healthcare, for which sharp is at full risk for hospital costs
			In-home medical consultation, ongoing evidence-based prognostication of further survival, caregiver support, and advance healthcare planning		
			**Activities and procedures**		
			***Setting care goals***		
			During the acute phase, registered nurses and social workers help patients and families set goals for medical and nursing care		
			***Regular phone calls and home visits***		
			During the acute phase, there are four to six weekly home visits from a registered nurse and one to three from a social worker. Once the goals of care are achieved, the patient enters the maintenance phase, the healthcare team continues to make regular home visits and phone calls		
			***Team training***		
			All members of the care team have received training on advanced illness and end-of-life needs		
Holley et al. (2009)	Palliative access through care at home (PATCH)	Patient chart review and caregivers	**Intervention focus**	Three geriatricians, one advanced practice nurse, and one social worker	Developed through the university of Chicago’s section of geriatrics and palliative medicine in 2006 and funded by a grant from the aetna foundation
			Improving patient quality of life and symptom management; reducing polypharmacy; supporting caregivers; and assisting with advance medical planning, complex decision-making, and transitions of care		
			**Activities and procedures**		
			***Home visit and care***		
			Four half-day home visits per week. Include primary care, palliative counseling, one-time in-home assessments, and emergency visits; frequency of visits varies from weekly to every three months, depending on the patient’s needs; four half-day home visits per week; participants have 24-h access to a member of the care team		
			***Collaborate with other professionals***		
			The senior registered nurse coordinates referrals and arranges for a variety of medical care while communicating with the patient, family members, pharmacy, home health agency, and other professionals		
Hum et al. (2020)	An integrated homecare program	People with advanced dementia were referred from the geriatric and palliative inpatient wards and outpatient clinics of a tertiary hospital	**Intervention focus**	A multidisciplinary team of doctors, nurses, and medical social workers	Temasek foundation cares for its funding of the palliative homecare program
			Person-centered care; psychosocial and spiritual support; optimal treatment of symptoms and comfort; educational support for caregivers; continuity of care		
			**Activities and procedures**		
			***Regular phone calls and home visits***		
			Conducting regular home visits and phone consultations		
			Assessing the patient’s needs during home visits to tailor the care plan accordingly		
			***Symptom management***		
			Designing personalized medication interventions to manage pain and behavioral issues such as agitation and restlessness		
			***Patient and family Education and support***		
			Providing education for caregivers, covering techniques like repositioning, environmental modifications, and the therapeutic benefits of music to alleviate discomfort		
			***Collaboration with referral hospitals***		
			Close collaboration with referral hospitals and inpatient hospices facilitates direct patient admission or allows for direct transition to ensure continuity of care		
Miranda et al. (2021)	Palliative home care	Nationwide administrative databases in Belgium (2010–2015)	**Intervention focus**	One general practitioner, two nurses and an administrative assistant	Funding comes mainly from social security contributions and taxes. All health insured persons in Belgium have a legal right to palliative home care support
			Advise general practitioners, health professionals, counsellors, informal caregivers, and volunteers involved in the provision of palliative home care of a patient and to organize and coordinate the provision of that palliative care at home between different care providers		
			**Activities and procedures**		
			The multidisciplinary team provides advice and support to patients and family caregivers		
			Palliative home care or physiotherapy		
			Setting allowance to cover costs associated with providing palliative care at home		
Pereira et al. (2023)	An integrated home-based palliative care program for advanced dementia (program dignity)	Geriatric inpatients and outpatients of a major Singapore tertiary hospital with advanced dementia; administrative databases of the recruiting hospital; case notes	**Intervention focus**	A multidisciplinary team of doctors, nurses, and medical social workers	A Singapore government-linked investment company contributed SGD$2 million
			Training of healthcare team members, ensure continuity of care, and providing support and education to family caregivers		
			**Activities and Procedures**		
			***Team Training***		
			Healthcare team members participate in courses related to dementia disease trajectory, end-of-life care, and various comfort measures		
			***Regular phone calls and home visits***		
			Nurses schedule regular weekly or bi-weekly phone consultations and home visits to monitor the patient’s condition. Additional home visits are arranged as needed		
			Home visits, conducted by a multidisciplinary team, typically last 2 hours		
			***Symptom management***		
			The team utilizes pharmacological and non-pharmacological measures to assess and manage patient symptoms		
			***Patient and Family Education and support***		
			Training is provided to family caregivers on handling patient symptoms		
			Medical social workers offer psychosocial and emotional support and assist in the application process for financial aid		
			***Collaboration with Other Professionals***		
			The team collaborates closely with other professionals to ensure appropriate medical care during the late stages of the disease. To avoid unnecessary hospitalization, ensuring the continuity of care		
Sternberg et al. (2019)	Maccabi Healthcare Services homecare program	Nurse case managers of the usual homecare program	**Intervention Focus**	A multidisciplinary team of doctors, nurses, spiritual care providers, and social workers	The project was funded by the Maccabi Foundation
			Training of medical team members, care continuity, and providing support and education to family caregivers		
			**Activities and Procedures**		
			***Time***		
			Implemented a one-year plan allowing individuals to participate for up to six months		
			***Team Training:*** Conducted two workshops to enhance team communication skills and deepen understanding of dementia and end-of-life dementia issues		
			***Review care Essentials***		
			Reviewed key points in palliative care and end-of-life, emphasizing the clarification of care goals, pain assessment, and legal frameworks		
			***Regular phone calls and home visits***		
			Provided round-the-clock telephone services with additional visits as needed to ensure continuous support		
			***Patient and Family Education and support***		
			Conducted bi-weekly team meetings to review patient conditions and care plans		
			Nurses explained the significance of end-of-life care, developed care plans, and offered ongoing guidance to family caregivers. Prepared toolkits for each family containing necessary items for home care. Spiritual care providers offered psychological and emotional support to people with dementia and their families		
Tay et al. (2020)	Palliative homecare program	Patients were recruited from a palliative homecare program	**Intervention Focus**	A multidisciplinary team of doctors, nurses, and medical social workers	Temasek Foundation Cares for its funding of the palliative homecare program. Subsequently, the Singapore Ministry of Health took over the funding of the program by subsidizing up to 80% of the program cost
			Advance Care Planning, team member training, care continuity, and family support		
			**Activities and Procedures**		
			***Team Training***		
			Healthcare team members participate in courses related to geriatrics and palliative care		
			***Advance Care Planning Workshops***		
			Conduct advance care planning workshops to promote the knowledge and skills of health care team members to discuss goals of care with family members		
			***Regular phone calls and home visits***		
			The nurse schedules regular telephone contact and home visits to assess the patient’s condition		
			Home visits are made once or twice a week for complex cases. Assess patients’ symptoms and quality of life using dementia-specific tools		
			***Psychosocial Support by Social Workers***		
			Social workers provide psychological and emotional support and assist in applying for financial aid when necessary		
			***Multidisciplinary Team Meetings***		
			Weekly multidisciplinary team meetings are held to review and discuss new complex cases, ensuring comprehensive attention to patients		
			***Collaboration with Referral Hospitals***		
			Close collaboration with referral hospitals and inpatient hospices facilitates direct patient admission or allows for direct transition to ensure continuity of care

**Table 4.** Home-based end-of-life care interventions mapped to the EAPC White Paper domains.

	Domain 1	Domain 2	Domain 3	Domain 4	Domain 5	Domain 6	Domain 7	Domain 8	Domain 9	Domain 10	Domain 11
	Applicability of palliative care	Person-centred care, communication and shared decision making	Setting care goals and advance planning	Continuity of care	Prognostication and timely recognition of dying	Optimal treatment of symptoms and providing comfort	Avoiding overly aggressive, burdensome, or futile treatment	Psychosocial and spiritual support	Family care and involvement	Education of the health care team	Societal and ethical issues
Brain Cassel et al. (2016)	✔	✔	✔	✔	✔	✔		✔	✔	✔	✔
Holley et al. (2009)		✔	✔	✔		✔		✔	✔		✔
Hum et al. (2020)		✔		✔		✔	✔	✔	✔		
Miranda et al. (2021)	✔	✔		✔					✔	✔	✔
Pereira et al. (2023)		✔	✔	✔		✔		✔	✔	✔	
Sternberg et al. (2019)		✔	✔			✔		✔	✔	✔	✔
Tay et al. (2020)		✔	✔	✔		✔		✔	✔	✔	

It is important to note that due to the lack of detail in the reporting of care interventions across studies, there is little or no identifiable supporting evidence for several domains of intervention for optimal palliative care for people with dementia, which included applicability of palliative care, avoid overly burdensome treatment, prognostication, and timely recognition of dying. In addition, few studies (Sternberg et al., 2019) reported the timing and duration of home-based end-of-life care for people living with dementia. Only one study (Sternberg et al., 2019) limited the time of end-of-life care, and patients were only allowed to participate in the home-based end-of-life care program for a maximum of six months.

### Description of home-based end-of-life care outcomes

#### Outcomes related to people with dementia

##### Quality of life

Only one study reported quality of life outcomes in people living with dementia after home-based end-of-life care (Hum et al., 2020). Findings reported that comprehensive palliative home care increased patients’ quality of life. Specifically, the median Quality of Life in Last-stage Dementia (QUALID)score decreased significantly from 23 (IQR 19–30) to 19 (IQR 15–24), *p* = .001 (see Table 5).

**Table 5.** Outcomes of home-based end-of-life care interventions for people with dementia.

	Outcomes related to people with dementia									Outcomes related to family caregivers		
	Quality of life	End-of-life comfort	Symptoms at the end of life	Healthcare service utilization	Costs	Place of death	Live discharge or long length of stays	Non-palliative interventions	Survival	Caregiver burden	Satisfaction with care	Grief
Bolt et al. (2019)											✔	
Brain Cassel et al. (2016)				✔	✔							
Holley et al. (2009)						✔					✔	
Hum et al. (2020)	✔		✔							✔		
Luth et al. (2021)							✔		✔			
Miranda et al. (2021)				✔	✔	✔						
Mitchell et al. (2004)			✔	✔				✔				
Mogan et al. (2022)											✔	
Pereira et al. (2023)				✔	✔							
Sternberg et al. (2019)			✔							✔	✔	
Tay et al. (2020)		✔									✔	
Treloar et al. (2009)					✔							✔

##### End-of-life comfort

Only Tay et al., (2020) assessed the comfort of dying during end-of-life care in the home for people living with dementia. This study found an improvement in comfort at the end of life for this population at home (*p* = .001). Notably, emotional distress and well-being exhibited marked improvements, with patients’ scores increasing from 11.4 (1.1) to 11.8 (0.6), *p* < .0001, and from 7.5 (1.8) to 8.0 (1.6), *p* = .007, respectively (Tay et al., 2020).

##### Symptoms at the end of life

Three studies examined the impact of home-based end-of-life care on symptoms in people living with dementia (Hum et al., 2020; Mitchell et al., 2004; Sternberg et al., 2019). Specifically, Hum et al. (2020) revealed a positive impact on reducing behavioral severity and improving nutritional status and pain (*p* < .05; *p* = .001; *p* < .05) (Hum et al., 2020). Sternberg et al. (2019) also supported this positive impact (*p* < .001) (Sternberg et al., 2019). However, Mitchell et al. (2004) found that pain symptoms and shortness of breath symptoms were less commonly reported by patients living in nursing homes relatively to those receiving home-based end-of-life care (37.1 % vs. 53.4 %, *p* < .001; 2.7% vs. 29.7%, *p* < .001). This may be partially attributable to the fact that patients living in nursing homes are relatively less exposed to pain-causing opportunities due to functional impairment and reduced mobility (AOR, 1.46; 95% CI, 1.04–2.03). Additionally, the use of oxygen therapy was more common in the nursing home setting (24.4% vs. 12.5%, *p* < .001) (Mitchell et al., 2004).

##### Healthcare service utilization

Four studies demonstrated significant positive effects of home-based end-of-life care interventions in reducing health care service utilization including reduced inappropriate medication use, surgeries, hospitalizations, admissions to the intensive care unit, and deaths in the hospital (*p* < .001) (Brian Cassel et al., 2016; Miranda et al., 2021; Pereira et al., 2023). In particular, Mitchell et al. (2004) also found that nursing home patients were more likely to be hospitalized compared to people with dementia receiving home-based care (31.5% vs. 43.7%, *p* < .001) (Mitchell et al., 2004).

##### Costs

Four studies examined the cost of providing end-of-life care for people living with dementia at home and all strongly support its potential for reducing healthcare costs (Brian Cassel et al., 2016; Miranda et al., 2021; Pereira et al., 2023; Treloar et al., 2009). Brian Cassel et al., (2016) study highlighted that this was due to patients who received home end-of-life care interventions requiring fewer hospitalizations and lower hospitalization costs, thereby reducing overall healthcare costs (Brian Cassel et al., 2016).

##### Place of death

Two studies have used place of death as an indicator to assess the effectiveness of home-based end-of-life care for people living with dementia (Holley et al., 2009; Miranda et al., 2021). The results of one study showed that people with dementia who used home end-of-life care support were more likely to choose to spend the last stages of their lives at home (75.7% vs. 32.6%; RR = 6.45) (Miranda et al., 2021). This finding is supported by another study (Holley et al., 2009).

##### Live discharge or long length of stays

Patients live discharged from hospice to seek treatment, or the condition of patients is stable and no longer eligible for hospice services, is known as “live discharge” (Russell et al., 2017). “Long length of stays” means long hospice stays exceeding 180 days (Luth et al., 2021). Only one study had explored live discharge and long length of stays in people with dementia during end-of-life care (Luth et al., 2021). Results showed that 39% (*n* = 1485) of people with dementia experienced a live discharge or long length of stays during end-of-life care, while patients receiving end-of-life care at home were more likely to experience live discharge or long length of stays compared to those in nursing homes (30% vs. 26%, *p* < .001).

##### Non-palliative interventions

Findings from one study (Mitchell et al., 2004) suggested that home end-of-life care programs appear to favor less invasive forms of care that may be more focused on patient comfort and quality of life. Findings (Mitchell et al., 2004) indicated that 27.2% of nursing home residents with dementia ultimately died from nonpalliative care measures of feeding tubes. In contrast, among people with advanced stages of dementia at home, this percentage was only 11.9% (*p* < .001).

##### Survival

One study focused on exploring the survival rates of people who received home-based end-of-life care compared to nursing home end-of-life care settings (Luth et al., 2021). The study showed that the predicted probability of survival for home hospice patients was higher than nursing home hospice patients at both 30 days and 90 days in hospice (0.83 vs. 0.81; 0.67 vs. 0.63). In addition, patients who received home-based end-of-life care had a 23% lower expected risk of death (*p* < .001).

#### Outcomes related to family caregivers of people with dementia

##### Caregiver burden

Two studies assessed caregiver burden when providing end-of-life care to people living with dementia at home (Hum et al., 2020; Sternberg et al., 2019), and provided inconsistent results. One of the studies indicated that family caregivers experienced mild to moderate burden (Hum et al., 2020). In contrast, another study showed that the home-based end-of-life care program had a positive effect on easing caregiver burden (*p* < .001) (Sternberg et al., 2019).

##### Satisfaction with care

Five studies reported outcomes of satisfaction with care (Bolt et al., 2019; Holley et al., 2009; Mogan et al., 2022; Sternberg et al., 2019; Tay et al., 2020). The results showed that not all studies consistently found that relatives of people living with dementia were satisfied with home end-of-life care. One qualitative study found that home care provided more personalized and emotionally supportive experiences compared to nursing homes (Bolt et al., 2019). In contrast, another study criticized home care services for failing to provide personalized end-of-life care, with an overemphasis on task-centered approaches and neglecting individual preferences due to resource and infrastructure inadequacies (Mogan et al., 2022). The generation of inconsistent findings also highlights the complexity of the field of home end-of-life care.

##### Grief

The existing literature on the impact of home-based end-of-life care on family caregivers’ grief is limited, with only one qualitative study providing relevant insights (Treloar et al., 2009). Specifically, family caregivers may alleviate their grief to some extent by taking on additional caregiving tasks and responsibilities, rather than simply delaying their grief experience. However, this study lacked further exploration of this result and failed to reveal in detail how family caregivers can regulate and relieve grief through their caregiving roles. In addition, the research results section lacks adequate quotations or specific examples to enhance its credibility, which to some extent limits the in-depth understanding of the research conclusions and may lead to misinterpretation or research bias. Therefore, in order to ensure the credibility and authority of the findings, further comprehensive studies are needed to validate this finding and remove potential biases.

## Discussion

### Main findings

This review systematically summarizes the existing evidence on home-based end-of-life care for people living with dementia, offering valuable insights into its viability as an end-of-life care option. Consistent evidence demonstrated from the findings is that home-based end-of-life care for people living with dementia reduces healthcare utilization and costs by decreasing the frequency and length of hospitalization, which is supported by the findings of previous studies (Brumley et al., 2007; Gomes et al., 2013; Gonzalez-Jaramillo et al., 2021). However, the advantages of home-based end-of-life care in terms of the overall cost of care for people living with dementia are uncertain. Due to the current focus of research primarily on assessing healthcare costs. This limited perspective prevents a full understanding of the overall costs of home-based end-of-life care. Candy et al. (2011) have suggested that if the cost analysis of end-of-life care at home encompassed all societal costs, then the cost differences between types of care might be smaller (Candy et al., 2011). For instance, the time and expenses associated with home visits may offset the cost savings from reduced healthcare utilization (Smith et al., 2024).

The place of death for terminally ill patients is often the focus of home-based end-of-life care (Shepperd et al., 2021). The findings of this review support that home-based end-of-life care increases the likelihood of people with dementia dying at home. However, dying at home is not considered a quality indicator of good end-of-life care (O'Leary et al., 2017), consistency between preferred and actual place of death is a better quality indicator (Hammer et al., 2023; Mansoor et al., 2023). Patients and caregivers participating in home-based end-of-life care programs suggests a strong inclination towards spending final days at home (Holley et al., 2009). This suggests that the outcome of the place of death is largely influenced by voluntary choices made by patients or their families. However, it also indicates that home-based end-of-life care can help people with dementia realize their wish to die at home.

While this review acknowledges the benefits of home-based end-of-life care on pain and symptom management, the results suggest that nursing home settings may better meet the needs of people living with dementia in this regard. Nursing home settings provide greater access to medical and nursing resources, which facilitates more effective management of pain and symptoms, such as oxygen therapy is more common in this setting (Mitchell et al., 2004). Despite pain and symptom management are recognized as crucial aspects of end-of-life care for people with dementia (McCaffrey et al., 2016), fewer reports of pain and symptoms in this review could indicate gaps in assessment and research coverage concerning these aspects for people with dementia. Indeed, existing literature suggests that pain is prevalent among people living with dementia during the end-of-life stage, but is often underdetected and undertreated (Thakur et al., 2017). This gap in comprehensive research on pain and symptom management during the home-based end-of-life care for people living with dementia limited understanding and response to this critical aspect.

Significantly, although home-based end-of-life care shows positive effects on people living with dementia, its effectiveness in supporting family caregivers remains inconclusive. Specifically, controversial findings exist regarding family caregivers’ burden and satisfaction. While some evidence suggests caregivers feel safer with the support of home-based end-of-life care services (Sarmento et al., 2017), they encounter numerous challenges in actual implementation. Limited face-to-face care from hospice workers often leaves family members as primary caregivers when workers are unavailable (Luth et al., 2021). Rowland et al. (2017) indicates patients in home-based end-of-life care required nearly 10 hours of care per day from family caregivers in their final three months, without which many patients would not be able to die at home (Rowland et al., 2017). Furthermore, a review finds that even with support, family caregivers inevitably face emotional and psychological stress (Becqué et al., 2019). Caregivers may feel pressure from professionals to act as professionals rather than loving relatives (Neergaard et al., 2008).

The review also examined the characteristics of home-based end-of-life care interventions for people living with dementia. Several elements of optimal palliative and end-of-life care are also well represented in this review of studies on end-of-life care at home for people living with dementia, as noted in the EAPC white paper (Van der Steen et al., 2014), although no single intervention can comprehensively address all of the 11 areas of optimal palliative and end-of-life care. In contrast to previous findings (Kochovska et al., 2020), this review suggests that current research placed a widespread emphasis on the continuity of care. This may reflect an improving focus on end-of-life care in the home for people with dementia and the recognition that transition to hospital may sometimes be in the best interests of the patient (Kochovska et al., 2020). However, the applicability of palliative care, avoiding overly treatment, prognostication, and timely recognition of dying has rarely been considered an area of interest by current research. This may be due to the uncertainty and difficulty in predicting survival, as well as the focus of care interventions on addressing unmet needs in this population rather than being model-driven (Kochovska et al., 2020). Davies et al. (2017) proposed that high-quality end-of-life care for people living with dementia does not need to be highly complex but should be tailored to individual needs (Davies et al., 2017). Furthermore, Round-the-clock access to care is considered a key component to be addressed when developing home-based end-of-life care services (Malcolm et al., 2020; Shepperd et al., 2021). However, it appears that the studies included in this review did not take this critical component into account, which also suggests potential areas for further research and development in home-based end-of-life care for people living with dementia.

Although some attributes of home-based end-of-life care for people with dementia have been described, the process of identifying best practices remains complex and elusive. As a previous systematic review of end-of-life care (Malcolm et al., 2020), there are few well-designed randomized controlled trials in this filed. The lack of high-quality randomized controlled trials hampers the identification of definitive, widely recognized best practice methods or models (Kaur & Li, 2024). Most of the studies included in this review were non-randomized controlled studies (Brian Cassel et al., 2016; Luth et al., 2021; Miranda et al., 2021; Mitchell et al., 2004; Pereira et al., 2023), these prospective and retrospective studies often reflect real-world rather than experimental situations (Saturni et al., 2014). Therefore, these studies can also provide valuable insights into service delivery. Unfortunately, these studies often lack comprehensive descriptions of the content of end-of-life care services and how service is delivery in practice, thereby introducing uncertainty into the actual implementation of care models. As noted previously, the lack of comprehensive descriptions of these interventions also hinders the ability to establish meaningful comparisons (Smith et al., 2014).

### Applicability, completeness and quality of the evidence

Because of the academic challenges within pertaining to the terminologies of palliative care and end-of-life care in this field, these studies used the broader term “palliative therapy” instead of “end-of-life care”, although the interventions in these studies exhibited characteristics of the end-of-life care service (Brian Cassel et al., 2016; Holley et al., 2009; Pereira et al., 2023). This issue appears to be a common occurrence in research within the palliative and end-of-life care domains (Malcolm et al., 2020), potentially leading to ambiguity in the interpretation and application of study findings.

Furthermore, it is necessary to interpret the findings of this review considering certain limitations. Due to ethical and methodological challenges (Akdeniz et al., 2021; Chen et al., 2014; Sampson et al., 2018), the number of studies eligible for inclusion in this review is relatively limited. This limitation resulted in many key outcome indicators receiving attention from an extremely limited number of studies. For example, quality of life, end-of-life comfort, survival, non-palliative interventions, and grief among family caregivers are all areas where only one study has focused (Hum et al., 2020; Luth et al., 2021; Mitchell et al., 2004; Tay et al., 2020; Treloar et al., 2009). While the review finds these results all demonstrate positive outcomes, some marginally significant outcomes may be the consequence of chance. For example, the results of a previous review clearly indicated that end-of-life care at home did not have an impact on caregivers’ grief (Gomes et al., 2016).

It is also important to note that many factors contributed to the heterogeneity of the results. In particular, some retrospective reporting may be affected by recall bias after the patient’s death, family members’ recall aspects are more generally limited (Song et al., 2016). Studies conducted at different times after patient death also contribute to heterogeneity in results (Claxton-Oldfield et al., 2010). Because patient end-of-life outcomes are more likely to be proxy-reported by family caregivers, the goals and values of family members are subject to change with time, resulting in their inability to accurately recall perceptions at a given point in time, which generates variability in measurement outcomes (Ernecoff et al., 2021). The diversity in study designs, outcome measurements, and the complexity of instruments used for outcome assessment in the studies included in this review also contributed to the heterogeneity of the study results. As a result, effectiveness assessments of home-based end-of-life care services has many methodological limitations, which hindered the ability to provide a definitive answer on the effectiveness of home-based end-of-life care services for people with dementia.

The absence of clear interventions and the variability in outcome measures also posed challenges in establishing associations between specific end-of-life care processes and outcomes in this review. These factors hindered the comparison of outcomes and synthesis of evidence, potentially impacting the generalizability of the findings. As described in previous studies the existing evidence base is too weak to robustly assess the effectiveness of home-based end-of-life care interventions for people with dementia (Miranda et al., 2019). Consequently, drawing accurate conclusions about the evidence of end-of-life care at home for people living with dementia proved to be a challenging task. Indeed, the lack of certainty in the evidence reflects the difficulties of conducting research in this area. Many studies examining home hospice and palliative care have struggled to draw specific conclusions (Hammer et al., 2023; Miranda et al., 2019; Shepperd et al., 2021).

Although the evidence supporting home-based end-of-life care for people living with dementia is limited, most of the studies reviewed reported positive results. This supports the intuitive hypothesis that home-based end-of-life care is beneficial for people with dementia, and that home-based end-of-life care services provide better opportunities for this population who want to die at home.

### Implications for research and practice

This review provides multiple insights that point to current research and evidence gaps in home-based end-of-life care for people living with dementia. To promote the development of this field, it is imperative for future studies to focus on the following aspects. Firstly, there is an urgent need for more research to develop and evaluate home-based end-of-life care services for people living with dementia. Considering the ethical challenges in conducting randomized controlled trial studies can be complex, future study designs such as case control studies could prioritize large samples with carefully matched intervention and control groups. Furthermore, future research endeavors to prioritize the utilization of consistent, standardized, and validated outcome measures are used to ensure accuracy and comparability of findings. Finally, it is crucial to consider the perspectives and needs of people with dementia, family caregivers, and healthcare professionals when delivering home-based end-of-life care. Addressing these stakeholders’ expectations will not only improve care services but also advance research in optimal home-based end-of-life care for people living with dementia.

It is also worth considering that the current lack of research may reflect a broader reality that while remaining at home until the end of life is often idealized, many people with dementia either choose not to stay at home at the end of life (Gott et al., 2004) or are forced to transition to other care settings due to the increasingly complex and demanding nature of their care needs (Seamark et al., 2014). The progressive nature of dementia often requires a level of intensive support and specialized medical attention that may surpass the capacity of home environments, even with family involvement (Mogan et al., 2018). This potential disconnect between the idealized vision of home-based end-of-life care and the practical realities faced by people with dementia and their families highlights an area worthy of further research. Consequently, future studies should also explore the barriers and facilitators that affect home-based end-of-life care for people with dementia, which could further illuminate the intricacies of providing effective home-based end-of-life care and is critical to improving care outcomes and advancing the overall understanding of optimal home-based end-of-life care for people living with dementia.

### What this review adds?

This review adds new insights into home-based end-of-life care for people with dementia. This review highlights that feasible and favorable outcome associated with such care, including reduced healthcare service utilization and costs, along with increased opportunities for patients to spend their final days at home. While the findings of this review consistent with previous systematic reviews (Candy et al., 2011; Grossoehme et al., 2020; Shepperd et al., 2021), this consistency is not surprising because it reflects the consensus in the field. But this review is unique in that it focuses on people living with dementia, drawing on the latest literature to shed light on the interventions and effectiveness of home-based end-of-life care services for people with dementia, filling a gap in previous evaluations of home-based end-of-life care services for this population. In addition, to provide a comprehensive assessment of home-based end-of-life care for people with dementia by combining quantitative and qualitative evidence, thereby adding variety and depth to the findings. This review also points out the limitations of existing research on home-based end-of-life care for people living with dementia and clear directions for future research.

## Conclusion

Home-based end-of-life care services provide better opportunities for people with dementia who want to die at home. However, interpretation of the evidence for home-based end-of-life care interventions for people living with dementia is constrained by the number of studies and methodological limitations. Additionally, the ambiguity of the study interventions and the heterogeneity of the outcome measures and outcome metrics, further complicate the interpretation of results. Therefore, although this review found other positive outcomes for home-based end-of-life care for people living with dementia, it cannot be conclusively assumed that these findings offer a definitive answer to the impact of home-based end-of-life care services. Given the urgent need for home-based end-of-life care for people with dementia and their family caregivers, there is still a need for more rigorous research to generate stronger evidence.
